# Estrogen/ER in anti-tumor immunity regulation to tumor cell and tumor microenvironment

**DOI:** 10.1186/s12935-021-02003-w

**Published:** 2021-06-07

**Authors:** Tiecheng Wang, Jiakang Jin, Chao Qian, Jianan Lou, Jinti Lin, Ankai Xu, Kaishun Xia, Libin Jin, Bing Liu, Huimin Tao, Zhengming Yang, Wei Yu

**Affiliations:** 1 Department of Orthopedics, Shengzhou People’s Hospital, #666 Dangui Road, Shengzhou, 312400 Zhejiang People’s Republic of China; 2 Department of Orthopedics, 2nd Affiliated Hospital, School of Medicine, Zhejiang University, #88 Jiefang Road, Hangzhou, 310009 Zhejiang People’s Republic of China; 3 Orthopedics Research Institute of Zhejiang University, No. 88, Jiefang Road, Hangzhou, 310009 People’s Republic of China

**Keywords:** Estrogen, Estrogen receptor, Tumor immunity, Tumor microenvironment, Cell differentiation

## Abstract

As the essential sexual hormone, estrogen and its receptor has been proved to participate in the regulation of autoimmunity diseases and anti-tumor immunity. The adjustment of tumor immunity is related to the interaction between cancer cells, immune cells and tumor microenvironment, all of which is considered as the potential target in estrogen-induced immune system regulation. However, the specific mechanism of estrogen-induced immunity is poorly understood. Typically, estrogen causes the nuclear localization of estrogen/estrogen receptor complex and alternates the transcription pattern of target genes, leading to the reprogramming of tumor cells and differentiation of immune cells. However, the estrogen-induced non-canonical signal pathway activation is also crucial to the rapid function of estrogen, such as NF-κB, MAPK-ERK, and β-catenin pathway activation, which has not been totally illuminated. So, the investigation of estrogen modulatory mechanisms in these two manners is vital for the tumor immunity and can provide the potential for endocrine hormone targeted cancer immunotherapy. Here, this review summarized the estrogen-induced canonical and non-canonical signal transduction pathway and aimed to focus on the relationship among estrogen and cancer immunity as well as immune-related tumor microenvironment regulation. Results from these preclinical researches elucidated that the estrogen-target therapy has the application prospect of cancer immunotherapy, which requires the further translational research of these treatment strategies.

## Background

The immune response differs with male and female. It has reported that females have the advantage in antimicrobic immunity and anti-tumor immunity, while females also suffer from the higher susceptibility to the autoimmune diseases (AD) [1]. Except for physiological structure variation on male and female, the gender-based immunity diversity is showed to account for the difference of sexual hormone secretion, such as estrogen, progestogen and androgen. However, the function of estrogen in cancer research still emphasized on the cancer cell proliferation [2], angiogenesis [3], epithelial–mesenchymal transition (EMT) [4], while the estrogen-induced cancer immune response alternation remains unclear. This review emphasized on the cell modification induced by estrogen and its receptor, estrogen receptor (ER), in canonical and non-canonical manner. On the other hand, the estrogen-related anti-tumor immunity regulation in different tumor microenvironment (TME) components was elucidated, including cancer cells, T cells, tumor-associated macrophages (TAM), cancer-associated fibroblasts (CAFs), and collagen in tumor tissues. These results suggested the potential for estrogen and ER as the therapeutic target in the cancer immunotherapy.

## Estrogen and estrogen receptor

Estrogen, which is mainly produced by the ovary and placenta of female animals, acts as a critical hormone that promotes the development of secondary sexual characteristics and maturation of sexual organs [5]. The biosynthesis relies on the function of CYP19a1, also called aromatase, performing the conversion from testosterone to estrogen [6]. The study found that the expression of CYP19a1 exists in different tissues in human, including liver, muscle, placenta, bone, breast, adipose tissue, and brain [7, 8]. So, it is not surprised that the sites of estrogen production are widely distributed over the different human organs and tissues. Multiple types of estrogen participate in estrogen metabolic process, including 13 known estrogen metabolites with different hydroxylation at the steroid ring, such as estrone, estradiol and estriol and so on [9]. CYP19a1 catalyzes the C19 aromatization of androstenedione and synthesizes the estrone. Then, estrone will consequently transform into estradiol and estriol. Among these estrogen types, 17β-estradiol (E_2_) is the most common estrogen form in human and has the potent function in estrogen pathway activation, which has the highest priority binding with estrogen receptor than estrone or estriol [10, 11]. E_2_ is mainly secreted by the ovaries and regulated by luteinizing hormone (LH) and follicle-stimulating hormone (FSH) during the menstrual cycle [12, 13]. The dramatic decay of E_2_ occurs in menopause while the concentration of estrone has only modest decline [14]. The alternation of estrogen causes the osteoporosis, senile vaginitis, cardiac symptoms and other menopausal syndrome.

The estrogen-induced targeted genes transcription relies on its binding with its receptor, called estrogen receptor (ER). The classical ER in mammal is divided into two distinct subtypes, ERα and ERβ, which are encoded by ESR1 and ESR2 and control the biological effects in vivo by recognizing estrogen in target tissues [15, 16]. These classical ERs mainly express in plasma membrane and nucleus, which contain a DNA-binding domain [17]. After activated by estrogen, the complex of estrogen and ER transfers to nuclear and locates to the estrogen response elements (ERE) in the distal enhancer regions of target genes [18]. Together with the coordinate regulators (p300/CBP, c-Jun, GATA3 and FOXA1), the activated ER enhancer regulates the genomic remodeling, including open chromatin architecture, histone modifications and trigger the ER-dependent genes transcription [19, 20]. On the other hand, the ER enhancer is able to connect with RNA polymerase II and promotes a series of noncoding enhancer RNAs transcription, called enhancer RNA (eRNA) [21]. The function of eRNA is still unclear. Some studies revealed that eRNA may regulate the gene transcription function by enhancing the strength of specific enhancer-promoter looping [22]. However, Rahman and his colleagues proved that eRNA-induced enhancer-promoter loops are unnecessary for the maintain of transcription by single-cell profiling [23]. Thus, the research in eRNA function in ER-induced regulation is a matter of considerable recent interest.

Besides the typical nuclear ER, it has been reported that membrane estrogen receptor (mER) also participated in the regulation of estrogen pathway. G Protein-Coupled Estrogen Receptor 1 (GPER1), also known as GPR30, is a member of G protein-coupled receptors located in on 7p22.3 chromosome, which is reported as a new membrane estrogen receptor in human [24, 25]. GPER1 has high affinity with estrogen and results to the rapid signal transduction by activating the cAMP, intracellular calcium and tyrosine kinase Src without DNA interaction [24]. Interestingly, Toran-Allerand found a novel mER called “ER-X”, which was mainly expressed in brain and uterus, especially in brain disorders. Although it shares some homology with ERα, the knock-out of ERα still has no influence on the expression of ER-X, suggesting the difference between ER-X and typical nuclear ER [26, 27]. However, until now, the ER-X is still unidentified, which requires further confirmed in the future.

## Signal pathway induced by estrogen in cancer

### NF-κB pathway

nuclear factor kappa-B (NF-κB) pathway controls the cytokine secretion, cell survival and proliferation, playing a critical role in inflammation development and immune regulation in TME [28, 29]. Estrogen receptor inhibits the expression level of Fos-related antigen 2 (Fra2) and down-regulates the NF-κB pathway. Both Fra2 and NF-κB synergistically promote the expression of RelB and Bcl-2, facilitating the EMT and bone metastasis in breast cancer [30, 31]. On the other hand, GPER enhances the phosphorylation of ERK and AKT and promotes the nuclear translocation of p65 as well as phosphorylation of IκB kinase β (IKK-β) after activation by specific GPER agonist, cadmium or G-1 [32, 33]. The phosphorylation and nuclear localization of NF-κB interact with IL-6 and contribute to angiogenesis and invasiveness in tumor progression, which can be reversed by G-1 [34]. However, the researches in breast cancer indicated that E_2_ reverses the anti-tumor effect of TNF-α by inhibiting NF-κB and further inactivating IAP, which blocks the process of tumor cells apoptosis [35]. Yang and his colleagues found that the knock-down of ERβ increases the expression of AKT and subsequently activates the phosphorylation of p65 in osteosarcoma. The ERβ-deficient osteosarcoma cells reveal a high level of proliferation, which can be reversed by PI3K inhibitor LY294002, indicating the key regulation in ERβ-induced PI3K-AKT- NF-κB pathway [36].

### MAPK–ERK pathway

Mitogen-Activated Protein Kinases (MAPKs), also known as extracellular signal-regulated kinases (ERKs), act as a regulator of cancer cell proliferation [37], differentiation [38] and metastasis [39]. The activation of ERK/MAPK depends on the stimulation of multiple growth factors, cytokines, viruses, G-protein-coupled receptor ligands and oncogenes, including epidermal growth factor (EGF), tumor necrosis factor (TNF), activators of protein kinase C (PKC) and Src family members [40]. In triple-negative breast cancer cell lines, MDA-MB-231 and MDA-MB-436, ER-α36 and EGFR are highly expressed while ER-α66 and ER-α46 are depleted. So, the treatment of E_2_ up-regulates the phosphorylation of ERK1/2 (p-ERK1/2) in dose-dependent manner and the decay of ER-α36 can reverse the E_2_-induced p-ERK1/2 activation [41]. Similar results are also reported in cervical cancer cells research, including CaSki and HeLa, which further confirmed the key function of ER-α36 in E_2_-induced ERK pathway activation [42]. The function of nuclear estrogen receptors also needs the mediation of insulin-like growth factor-1 (IGF-1). IGF-1R causes the mobilization of MAPK pathway and promotes the neuroestrogen synthesis and estrogen-induced targeted gene transcription, which is antagonised by PI3K [43]. On the other hand, the treatment of estrogen promotes the production of reactive oxygen species (ROS) and mediates the activation of p38 MAPK, which can be abolished by antioxidant, NAC. Furthermore, Estrogen-induced p38 MAPK enhances the expression of p21 in hypoxia and reduces the proliferation of breast cancer by G_1_/S phase cell cycle arrest, suggesting the proliferation adjustment in MAPK pathway induced by estrogen [44].

### β-catenin pathway

**Fig. 1:**
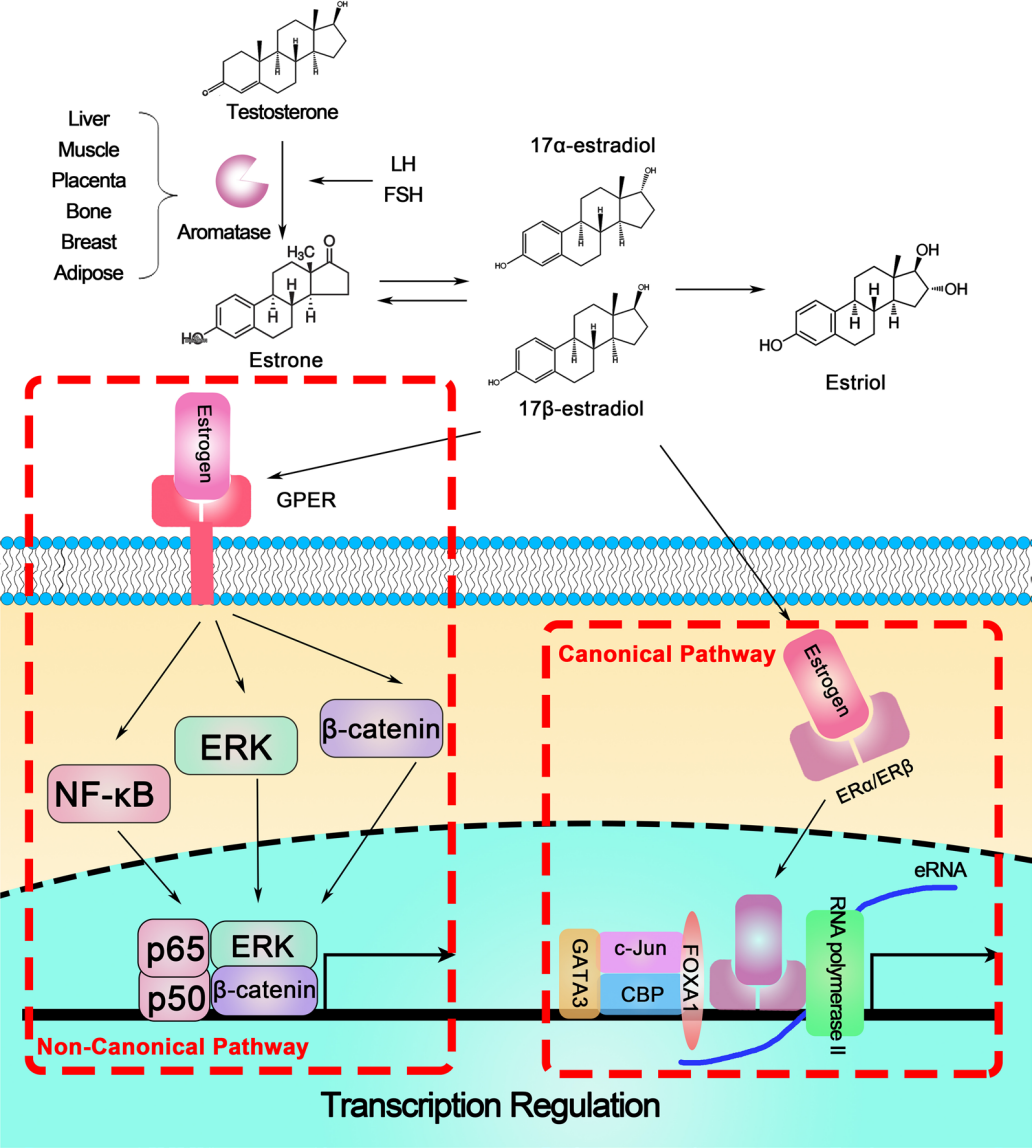
The synthesis and function of estrogen in cell transcriptional regulation. The aromatase expressed in liver, placenta, bone and breast promotes the synthesis of estrone from testosterone with the stimulation of LH and FSH. Then estrone mediate the further synthesis of estradiol including 17α- and 17β-estradiol and finally synthesize estriol by hydroxylation. Sequencely, estrogen alternate the immunological differentiation by two pathways. (1) Canonically, estrogen promotes the transcription of estrogen-target genes by ERα/β activation together with co-factor, including GATA3, c-Jun, CBP, and FOXA1. (2) Non-canonically, estrogen enhances the NF-κB, ERK and β-catenin pathway by GPER activation

The Wnt/β-catenin pathway plays an important role in development, tissue renewal, cell proliferation and differentiation, and its deregulation is associated with numerous diseases [45, 46]. Guo’s research found that the treatment of Bisphenol A, a weak estrogen agonist, promotes the expression of β-catenin and causes elevated expression of cancer stem cell markers, accelerating the human ovarian cancer cell-derived sphere formation [47]. In ER^−/−^ mouse colon tissue and colon cancer cells, the expression of Wnt2b, LRP8 and Dvl1 are significantly decreased, which are all the Wnt/β-catenin complex genes, similar with the result of the selective ERα antagonist treatment [48]. The Estrogen-induced β-catenin activation also depends on the assistant of Wnt3a and HDAC signaling pathways, which promotes the nuclear localization of β-catenin and enhance the transcription of β-catenin-regulated genes, including CCND1, c-Myc, and the androgen receptor (AR) [49]. Furthermore, in non-canonical pathway, the treatment of E_2_ interacts with the receptor of IGF-1 (IGF-1R) and sequentially activates the PI3K/Akt/GSK3β signaling with or without the IGF-1, promoting the stabilization of β-catenin and the transcription of the β-catenin-related genes in Wnt-independent pathway [50]. However, Kang’s research found that as a non-canonical estrogen receptor signaling, estrogen-related receptor gamma (ERRG) is highly expressed in gastric cancer and acts as an antagonizer, which induces the proteosome degradation of β-catenin and inhibits β-catenin-TCF4/LEF1 binding to the targeted genes [51, 52]. So, the estrogen-induced β-catenin regulation is still unclear, requiring further investigation (Fig. 1).

## Estrogen/estrogen receptor in cancer immunotherapy

It has reported that estrogen sex steroid hormones and its receptor present a comprehensive and various physiological functions in the process of tumor development [13]. The estrogen/ER-targeted endocrine therapy has been the standard therapy in breast cancer treatment, such as selective estrogen receptor modulators and aromatase inhibitors [53]. Meanwhile, the abnormal expression levels of estrogen receptor (ER) are also related to the susceptibility to various cancer and alternate the prognosis of tumor patients, especially those of the breast and endometrium [13, 54]. The estrogen-induced therapeutic outcome is also related to the function of anti-tumor immunity, especially in breast cancer, which is closely related to the expression of ER [55]. Abrahamsson and his colleagues found that the estrogen antagonists, tamoxifen and fulvestrant, inhibit the infiltration of neutrophils, fibroblast as well as M2 macrophage and increase the innate immune response in breast cancer [56]. Furthermore, the research from Márquez-Garbán designed a series of selective ER down-regulators (SERDs) and found that the application of SERDs suppression of myeloid-derived suppressor cells (MDSC) and synergistically increased the therapeutic effect of immune checkpoint inhibitors [57]. In this regard, it has a huge amount of potential in the combination between SERDs and immunotherapy. However, it variates in different patients because of the diversity of cancer types. Finding the specific regulation pathway to tumor immune response becomes the importance for the cancer immunotherapy.

## Cancer cell

### Immune check point

The expression of programmed death ligand-1 (PD-L1) on the surface of tumor cells interacts with its receptor in T cell, Programmed Death-1 (PD-1) and causes the dysfunction of T cell in tumor tissue, which inhibits the T cell-induced anti-tumor immunity. In eutopic epithelial cells, Wu’s research revealed that 17β-estradiol enhances the expression of PD-L1 in a dose-dependent manner [58]. In cancer cells, meanwhile, the bio-information data and cell lines researches showed that the expression level of PD-L1 in ERα^+^ breast cancer is inferior to the ERα^−^ breast cancer [59, 60]. The clinical data showed that female is a disadvantage factor in PD-1-induced melanoma therapy, indicating that the sex hormone from female has the key function in PD-1/PD-L1 axis regulation [61]. The Next Generation Sequencing (NGS) results confirmed that the treatment of E_2_ is involved in the transcription of PD-L1 in MCF-7 cell line, indicating the negative regulation of PD-L1 induced by estrogen in transcriptional level [62, 63]. The research reported that high level expression of ER enhances the expression of IL-17E, while the expression of IL-17A, IL-17C, and IL-17F is significantly inhibited. IL-17E acted as an antagonist of IL-17 pathway by suppressing the activation function of IL-17A, IL-17C, and IL-17F. The inhibition of ER-induced IL-17 signaling decreases the expression level of PD-L1 in ER^+^ breast cancer, which reveals the essential role of IL-17 in ER-induced PD-L1 expression decrease [64].

GPER signaling was reported the cross-talk with PD-L1, which contributes to the suppression of tumor immune escape [65, 66]. The treatment of GPER agonist G-1 decreases the cell proliferation of melanoma and suppresses the expression of PD-L1. Also, the combined therapies of anti-PD-1 antibody and G-1 effectively retard the process of melanoma [65]. The similar results also exist in pancreatic ductal adenocarcinoma treatment, which further showed that the G-1-induced inhibition of PD-L1 relies on the phosphorylation of RB (p-RB) and the expression of c-Myc [67]. It seems that NF-κB is the key regulatory pathway in GPER-induced PD-L1 down-regulation. The evidence indicated that NF-κB is decayed after the interaction between GPER and E_2_ [34]. The inactivation of NF-κB inhibits the expression of COP9 Signalosome Subunit 5 (CSN5), which can be reversed by GPER. CSN5 plays a critical role in the ubiquitination adjustment of PD-L1 and the down-regulation of CSN5 enhances the poly-ubiquitination of PD-L1 and promotes the degradation via ubiquitination pathway [35, 68].

However, the contradictory result from Yang’s research comes out that E_2_-induced PI3K/Akt pathway activation promotes the expression of PD-L1 by post-transcriptional control and inhibits the anti-tumor function of T cell in ER^+^ breast cancer [69]. On the other hand, the estrogen-induced ERK activation is also related to the expression alternation of PD-L1. The study found the mutual upregulation of PD-L1 and Bcl-2-associated athanogene-1 (BAG-1) via ERK signaling and caused the resistance of tyrosine kinase inhibitor (TKI) [70]. In addition, the chemotherapeutic treatments of esophageal squamous cell promote the phosphorylation of ERK, consequently enhancing the expression level of PD-L1 and responding for tumor metastasis [71]. As the agonist of ERK, the stimulation of E_2_ may promote the expression level of PD-L1 via ERK pathway in ER-positive breast cancer. These results suggested that estrogen can also enhance the expression of PD-L1, which is different from the previous research we have discussed before. So, to illuminate the specific function of E_2_/ER in PD-L1 regulation, the further researches are urgently required.

### Cancer stem cell

Cancer stem cells (CSCs) are one of special cancer cell subtypes, which have the ability to self-renew and differentiate to heterogeneous tumor cells [72]. The heterogeneity of CSCs causes the alternation of antigen on the surface of tumor cells and prevents the T cell-induced surveillance [73]. On the other hand, CSCs express high level of PD-L1, IDO1 and enhance the secretion of inhibitory chemokines, including IL-8, CCL2 and CCL5, which are related to the deplete of T cell and Treg differentiation [74, 75]. Uchiumi’s study found that the ER^+^ breast cancer cells express higher populations of CD44^+^/CD24^−^, revealing the high level of CSCs retaining ability. On the contrary, ER^−^ basal-like breast cancer cells showed the lower stemness in terms of the decrease of ALDH activity [76]. Meanwhile, the surface plasmon resonance test also illuminated that the treatment of estradiol promotes the migration of CSCs and facilitates the metastasis in colorectal cancer [77]. The research proved that the ER-induced DLL1 expression is the key factor in CSCs differentiation. In the ER^+^ luminal breast cancer, estrogen stabilizes the DLL1-induced Notch signaling activation and controls the function of CSCs. The knock-down of DLL1 reduces the expression of CD24 and CD44, which is the biomarker of breast cancer CSCs, and down-regulates both tumor growth and lung metastasis of luminal breast cancer [78]. Meanwhile, ERα and ERβ exhibit elevated expression of FGF and EGF in CSCs culture and the treatment of estrogen also promotes the expression of CSCs markers in a time-and dose-dependent manner in thyroid cancer, showing the estrogen-induced CSCs differentiation in growth factor-dependent manner [79]. However, Domenici and his colleagues found that ERα inhibits the SOX-2 and SOX-9, which are the biomarkers of CSCs in breast cancer, suggesting the complexity of estrogen-induced CSCs transforming [80]. Meanwhile, the similar results in osteosarcoma elucidate that decitabine promotes the expression of ERα induced by the decrease of DNA methylation and suppresses the biomarker of CSCs, including SOX2, OCT4, and NANOG [81]. This contradictory reveals the complicated regulation of estrogen/ER pathway to CSCs differentiation.

### T cell

Cytotoxic T lymphocytes (CTLs) are the principal force in anti-tumor immunity, which are regulated by heterogeneous phenotypes of helper T lymphocytes. Estrogen acts an important role in T cell development and maturity. Moulton found that the treatment of E_2_ leads to the drastic thymic atrophy [82]. This process can be reversed by decrease of endogenously produced estrogen after ovariectomy [83]. Meanwhile, E_2_ causes the down-regulation of thymic T cell number and inhibit the proportion of CD4 and CD8 double-positive T cells, while the level of CD4 or CD8 single-positive T cells and CD4^−^CD8^−^CD25^−^CD44^+^ T cells increase. The alternation of T cells maturity causes the inhibition of T cell dependent inflammation [84]. However, Adori and his colleagues found that estrogen regulates B and T cells by the activation of ERK and AKT phosphorylation, cell-specific Ca2^+^ signal, and NF-κB pathway, which enhance the T cell-dependent immune response, suggesting the diversity of estrogen-induced regulation in T cells [85].

Estrogen also adjusts the T cell differentiation in the tumor-infiltrating lymphocytes in tumor tissue. T regulatory cells (Treg) (CD4^+^ FoxP3^+^) and T helper (CD4^+^ FoxP3^−^) is significantly increased in ER mutant breast cancer, while CTLs had no difference, indicating the correction between ER and Treg differentiation [86]. With the stimulation of 17β-estradiol, ERβ promotes the differentiation of Treg, which dominates the secretion of IL-10 as well as TGF-beta and down-regulate the activity of CD8^+^ T cell, causing the suppression of T cell-induced tumor immunocytotoxicity [87, 88]. Garnier and his colleagues found that the E2-induced Treg differentiation in pregnancy-level concentrations also suppresses the differentiation of Th17 cells by the adjustment of PD-1/PD-L1 axis, which is elevated expressed on CD4^+^ T cells with the treatment of E_2_ [89, 90]. Moreover, the E_2_ also inhibits the function of antigen presenting cells (APC) and up-regulates the Treg activity assisted by bone marrow-derived dendritic cells [91]. However, the research also revealed that estrogen-induced Treg activation only existed in high level of estrogen. However, the result totally reversed in low estrogen level [92]. The similar result also showed that the differentiation of Th1 cells enhanced in low estrogen level, which promoted the Th1-induced CTL activation and up-regulated the anti-tumor immunity, while the high level of estrogen increased the proportion of Th2 phenotype, causing the immunity inhibition [58]. In this regard, estrogen indicated the bidirectional function in Treg differentiation, which has the different regulation in cancer immunity in a dose-dependent manner.

## Tumor-associated macrophage

As the essential regulator in tumor immunity, macrophages have disparate differentiation, which can generally be separated into two phenotypes: M1 and M2 macrophages [93]. Blood monocytes or tissue-resident macrophages are recruited and functional reprogrammed induced by multiple chemokines secretion, including CCL2, CCL5, C5a, CSF-1, VEGF and IL-34 [94]. The macrophages in tumor tissues, also called TAMs, constitutes the dominant component of the infiltrating leukocyte in all tumor, which are mainly differentiated into M2 phenotype [95]. TAMs are canonically associated with the immunosuppression by following points: (1) TAMs express high level of IDO1 and suppress the activation and proliferation of CD4^+^ and CD8^+^ T cells and promote the immunosuppression function of Treg through kynurenine pathway [96]. (2) TAMs secret the immunosuppression cytokines regulate the T cell-induced ant-tumor immunity, including IL-10, IL-6, PGE2, and TGF-beta1 [97]. (3) TAMs express PD-L1 and PD-L2 and participate exhaustion of CD8^+^ T cell by cell–cell interaction[98, 99].

Estrogen close ties to the polarization and function of macrophage, which has been widely researched in osteoporosis studies. Dou’s researches showed that the proportion of M1 increases in ovariectomized mice and the activation of ERα reduces the M1/M2 ratio by ERα selective agonist 4,4′,4″-(4-propyl-[1H]‐pyrazole-1,3,5-triyl) trisphenol (PPT) [100]. The loss of ERα in macrophages increases the expression of NOS2 and reduces the Arg1 expression, which indicated the M1 macrophages activation [101]. Jing and his colleagues reported that ERα in macrophages promotes the expression of chemokine (C–C motif) ligand 18 (CCL18) and accelerates the mTOR/KIF5B-induced EMT of endometrial cancer and cancer immune escape [102, 103]. Meanwhile, Côté’s study exhibited that GPER1 is also involved in the shift of macrophages toward M2 phenotype together with ERα by depleting the mobilization of NF-κB and iNOS pathway, also showing the participation of GPER in macrophages polarization [104]. The regulation of E_2_-induced M2 macrophages transformation in the cancer immunity mainly derived from the alternation of cytokine microenvironment. The ERα enhanced the secretion of pro-inflammatory cytokine by down-regulation the PI3K and Akt pathway activation, stimulating the TLR ligand in the surface of macrophages [105]. In response to TLR4 signaling in macrophages, the E_2_ mediated the secretion of inflammatory cytokine (IL-1β, IL-6, and TNF-α) and contributed to the tumor-associated inflammation and cancer immune escape [106, 107]. Overall, these findings suggested that estrogen decreases the M1 subtype and facilitates the M2 polarization of TAM, which is related to the suppression in anti-cancer immunity.

## Cancer-associated fibroblast

Similar with the macrophages, the fibroblasts in tumor tissue, CAFs, are also able to alternate anti-tumor immune. In the innate immunity, CAFs can escape from radiotherapy and suppress the function of Natural Killer (NK) cells by producing PGE2, IDO, TGF-β and other immunosuppressant compounds [108, 109]. Furthermore, CAFs aggravate the glucose deficiency in the milieu and inhibit the migration of cytotoxic T lymphocytes (CTLs) in adaptive immunity [110]. The expression of PD-L1 in CAFs also causes the depletion of CTLs and down-regulates the anti-tumor immunity [111]. By the expression of B7H3, CD73, and DPP4, CAFs maintain the differentiation of Treg and enhance the inhibition T cell proliferation [112]. Taken together, the proportion of CAFs in the tumor tissue is a foretaste of repression of cancer immunity.

The researches have elucidated that CAFs are related to the resistance of tamoxifen-induced endocrine therapy, indicating the relationship between CAFs and estrogen pathway [113]. The main estrogen receptor in breast CAFs is GPER. The activation of GPER by G-1, E_2_ or 4-hydroxytamoxifen (OHT) promotes the expression of vascular endothelial growth factor (VEGF), connective tissue growth factor (CTGF), c-fos, Cyr61, EGR1 and regulates the adhesion/spreading, proliferation and migration of CAFs in rapid and slow manner in breast cancer [114, 115]. The GPER-mediated CAFs proliferation results from the expression enhancement of FGFR1, which up-regulates the sensitivity of FGF2 and promotes the progression of breast cancer by paracrine manner [116]. CAFs up-regulate the secretion of serval growth factor with the treatment of estrogen, including E2, IL-1β1, VEGF and so on [117, 118]. These factors facilitate the EMT of breast cancer by hypoxia, COX-2 and ABCG2 transcription and facilitate the immunity escape [118, 119]. Moreover, estrogen promotes the secretion of IL-6 derived from CAFs, which acts as the immunosuppression cytokine and inhibit the cancer immunity [120, 121]. These findings suggested that E_2_ and ER is a critical regulator in CAFs-induced immune escape.

## Cytokines and chemokines

The interaction between cancer cells and immunity induced by cytokines and chemokines plays an important role in cancer initiation, development and therapeutic effect [122]. The researches have elucidated the E_2_-induced inflammatory cytokines secretion in tumor and immune cells, revealing another E_2_/ER-induced regulatory pathway in cancer immunity. The activation of ERβ by LY500307 promotes the secretion of IL-1β and enhance the infiltration of neutrophils in cancer tissue, which inhibits the tumor progress and lung metastasis [123]. Recent research found that the enhancement of estrogen facilitates the secretion of pro-tumor cytokines from Treg, Th2, CAFs and other immunosuppressive cells, including IL-4, IL-6, TNFα, and IL-17A while decrease the expression of anti-tumor cytokines from M1, NK, CD8^+^ T cells and Th1 cells, including IL-12 and IFNγ [124]. These estrogen-induced secretion pattern causes the M2 shifting and dominate the immunosuppression in TME. Taking tagether, these results reveal that E_2_/ER has the diversity effects on the cancer immunity which is according to the target cell types.

On the other hand, the expression of ER is related to the secretion of chemokines, which acts as the bridge between cancer cells and immune cells. The estrogen and ERα expression enhances the secretion of CCL2 and increases the expression level of macrophage-induced C-X-C Motif Chemokine Ligand 12 (CXCL12) [125, 126]. Meanwhile, ERα can also promote the CXCL11 secretion of cancer cells and activate EMT induced by CXCR7 [127]. The secretion of CCL2 and CXCL cytokine subfamily cause the infiltration and M2 conversion of macrophages in the cancer niche, leading to the immune escape and long-distance metastasis. In this regard, these researches indicated that the E_2_/ER-induced cytokines and chemokines secretion in different cellular compartments of TME can be the effective therapeutic target to optimize the cancer immunotherapy efficacy.

## Collagen and tumor stroma

The diversity of biology structural and biological functions attribute to the expression of collagen, which is the tissue and molecular scaffold of animal life [128]. The collagen is the dominating composition in cancer tissue, which is composed of the tumor stroma, providing the mechanical support to the cancer cells. The biosynthesis of collagen is manipulated by the TME, including secretion of TGF-β [129], FGF [130] and estrogen [131]. Collagen has the key function in anti-tumor immunity regulation, together with macrophages, lymphocytes, and fibroblasts [132]. The expression of collagen mediates the dysfunction of CD8^+^ T cell in LAIR1-dependent manner and depleting the LOXL2, the key factors in collagen synthesis process, reverses the suppression of T cell infiltration [133]. These results suggested the importance of collagen in cancer immunity regulation.

As an essential factor in collagen regulation, ERα promotes the transcription of miR-1271 in T47D and MCF-7 and regulates the expression of TGF-β, which is the essential cytokine in collagenous fiber synthesis and manipulates the alternation of cancer tissue fibrosis and immunity [134, 135]. The research revealed that collagen can be the biomarker of breast density, which has a positive correction with ERβ2 expression in breast cancer [55]. Huo and his colleagues also found that collagen expresses elevated in high mammographic density (LMD) breast tissues together with the significantly overexpression of aromatase, which revealed the highly production of estrogen. However, the expression of ERα and ERβ present no difference in LMD, suggesting the secretion of collagen is related to the stimulation of estrogen rather than the up-regulation of ERβ [136]. The specific mechanism of E_2_/ER-induced collagen synthesis in cancer tissues is possibly related to the MAPK pathway and the activation of AP-1, which can be blocked by tamoxifen [137]. However, the estrogen-induced tumor collagen synthesis still requires the further confirmed.

One possible reason for the estrogen-induced collagen immunosuppression is that the expression of collagen increases the density of tumor stroma, which reduces the infiltration of lymphocytes and avoids the contact between CTLs and tumor cells. In this process, the expression of stroma ColXα1 has proved the essential role in tumor-infiltrating lymphocytes regulation, which indicated the low overall survival in ER^+^/HER2^+^ breast cancer [138, 139]. On the other hand, the densified tumor stroma reduces the oxygen diffusion in tumor tissues and promotes the expression of hypoxia-inducible factor 1α (HIF1α), which induces immune checkpoint marker expression and immunosuppressive cytokines release [55, 140]. Moreover, the accumulation of collagen-I in mammary carcinomas promotes the transformation to CSCs through the activation of AKT-mTOR and YAP pathway and increases the possibility of cancer metastasis [141].

## Conclusion and prospection

**Fig. 2:**
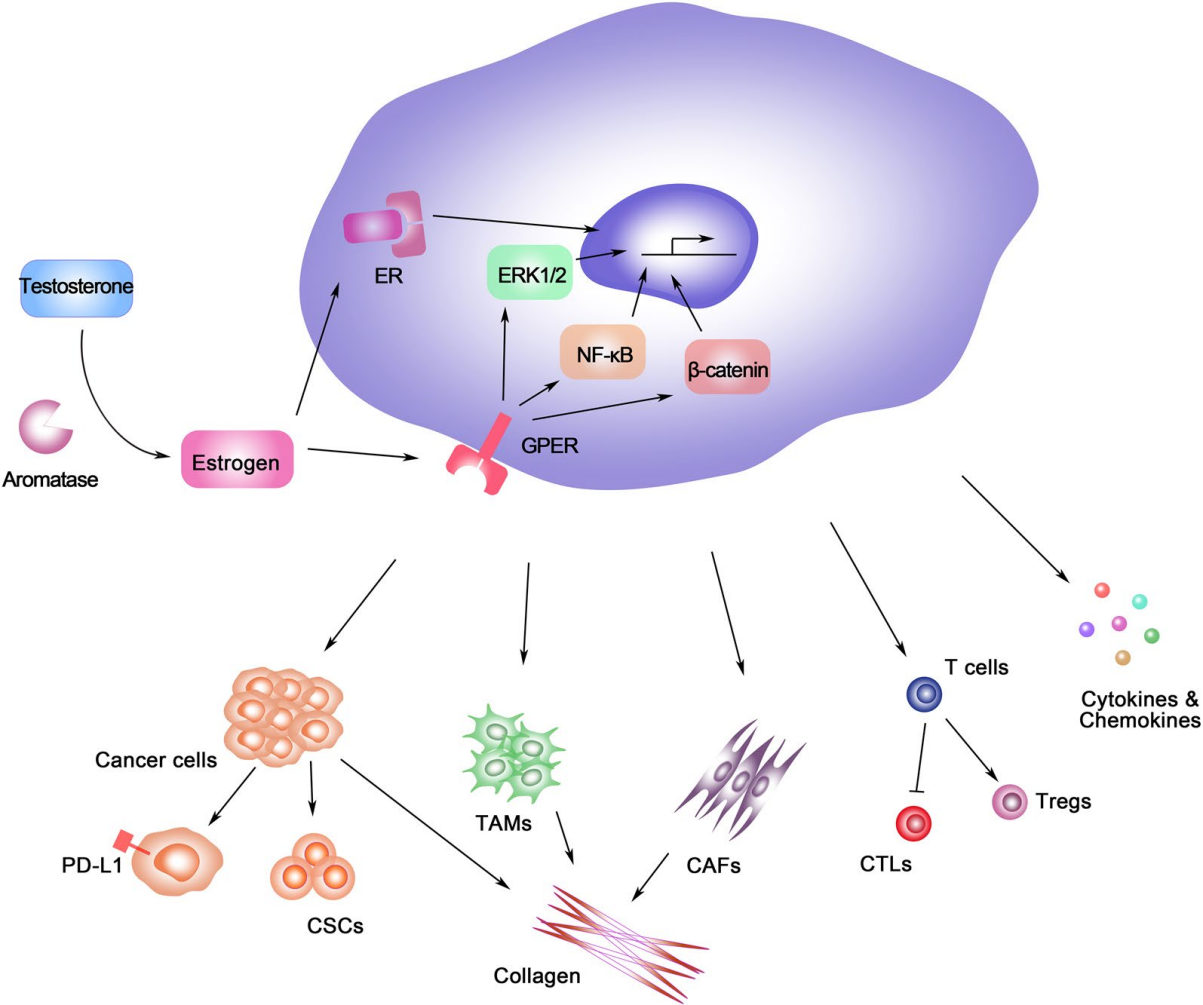
The schematic diagram of estrogen-induced anti-tumor immunity regulation. The estrogen-related immunity reugulation including: (1) mediate the expression of PD-L1 and CSCs differentiation; (2) TAMs differentiation and secretion; (3) CAFs proliferation; (4) T cell maturity and Treg regulation; (5) Cytokines and chemokines secretion regulation

The estrogen functions as the immune regulator in anti-tumor immunity in diverse pathways. This review mainly summarized the E_2_-related canonical and non-canonical cell activation signal pathway. Then the estrogen-induced immunologic differentiation in cancer cells was mainly elucidated, such as PD-L1 expression and CSCs regulation. Moreover, the estrogen-induced recruitment and chromatin remodeling of immune-related cells, including T cells, TAMs, CAFs, and the expression of cytokines and chemokines, collagen as well as the tumor stroma, is also discussed. The specific estrogen-induced regulation in cancer and TME was summarized in Fig. 2 and Table 1. In view of the regulatory function in this TME component, several potential combinations of estrogen/ER-target therapy for cancer treatment have been tested in various research, including the immune checkpoint inhibitor therapy [142], immune-modulating therapy [143], and cancer vaccination therapy [144]. Meanwhile, the therapies targeting to the estrogen-induced cytokines secretion, including IL-17, CCL2, FGF, TGF-β, et al. and consequent differentiation of cancer and T cells should also be taken into consideration to reverse the hormone-related cancer immunity depletion. Although it is still facing challenge, the further research in the estrogen-induced immunologic regulation will provide the foundation to illuminate the endocrine-related TME and optimize the immunotherapy in cancer treatment.

**Table 1:** The regulatory function of estrogen and ER in cancer cell and various TME components

TME	Subtype	Regulation	Mechanism	References
Cancer cells	PD-L1	Down	Decrease the PD-L1 transcription	[62]
			Inhibite the expression of PD-L1 by IL-17 pathway down-regulation	[64]
			Cause p-RB and c-Myc expression	[67]
			Enhance the poly-ubiquitination of PD-L1 by CSN5 in GPER-induced NF-κB pathway	[35, 68]
		Up	Promote the post-transcriptional modification of PD-L1	[69]
			Activate the ERK-induced PD-L1 expression (prediction)	[70, 71]
	CSCs	Up	Stabilize the DLL1-induced Notch signaling activation	[78]
			Mediate the elevated expression of FGF and EGF	[79]
		Down	Inhibit the expression of CSCs marker by DNA methylation decrease	[81]
T cells	CTLs	Down	Alternate T cell maturity	[82–84]
		Up	Activate the ERK and AKT phosphorylation, cell-specific Ca2^+^ signal, and NF-κB pathway	[85]
	Tregs	Up	Increase the differentiation of Treg	[86–88]
			Inhibit the function of APC	[91]
		Double-regulation	Treg increase in high estrogen level and decrease in low estrogen level	[58, 92]
TAMs	M1	Down	Cause the NOS2 repression and Arg1 increase	[101]
	M2	Up	Deplete the mobilization of NF-κB and iNOS pathway by GPER1	[104]
CAFs		Up	Promote the expression of VEGF, CTGF, c-fos, Cyr61, EGR1, and FGFR1	[114–116]
Cytokines & chemokines	Cytokines	Double-regulation	Increase IL-1β from cancer cells Increase IL-4, IL-6, TNFα, and IL-17A from immunosuppressive cells Decrease IL-12 and IFNγ from effector T cells	[123, 124]
	Chemokines	Up	Promote the secretion of CCL2, CXCL11, and CXCL12	[125–127]
Collagen		Up	Manipulate by MAPK pathway and AP-1 (prediction)	[128]

*TME* Tumor Microenvironment, *CSCs* cancer stem cells, *CTLs* cytotoxic T lymphocytes, *Tregs* regulatory T cells, *TAMs* tumor-associated macrophages, *CAFs* cancer-associated fibroblasts

## Data Availability

Not applicable.
